# A three-decade lake dataset on the Mongolian plateau tracking water area and quality dynamics (1990–2020)

**DOI:** 10.1038/s41597-025-06059-5

**Published:** 2025-11-14

**Authors:** Jinkai Guo, Kai Liu, Jiaming Na, Ge Liu, Zhigang Cao, Chenyu Fan, Bin Xue, Junchuan Huang, Chunqiao Song

**Affiliations:** 1 College of Civil Engineering, Nanjing Forestry University, Nanjing, 210037 China; 2 State Key Laboratory of Lake and Watershed Science for Water Security, Nanjing institute of Geography and Limnology, Chinese Academy of Sciences, Nanjing, 211135 China; 3 University of Chinese Academy of Sciences, Nanjing (UCASNJ), Nanjing, 211135 China; 4 Northeast Institute of Geography and Agroecology, Chinese Academy of Sciences, Changchun, 130102 China; 5 University of Chinese Academy of Sciences, Beijing, 100049 China; 6 Research Center of Applied Geology of China Geological Survey, Chengdu, 610036 China

**Keywords:** Limnology, Hydrology

## Abstract

Lakes on the Mongolian Plateau (MP) are vital freshwater and ecological resources, yet comprehensive long-term records of their area and water quality dynamics have been lacking. To fill this gap, we developed the Mongolian Plateau Lake Dataset (MPLD)—the first open-access, spatially explicit dataset providing annual records (1990–2020) of lake extent and three water quality indicators (Secchi Disk Depth, Total Suspended Matter, and Forel–Ule Index) for 1,161 lakes ( > 1 km², including artificial reservoirs). Built from over 56,000 Landsat scenes processed via Google Earth Engine, the dataset reveals a pattern of initial lake shrinkage followed by partial recovery, with total lake area reaching 19,592.78 km² by 2020. By that year, 71.83% of lakes were classified as eutrophic (FUI ≥ 10).The data show strong spatiotemporal heterogeneity: western and northern lakes expanded and cleared, while eastern and central Inner Mongolia saw contraction and water quality decline. The MPLD provides critical support for water resource management across the MP and serves as a valuable reference for global studies on climate change and dryland dynamics.

## Background & Summary

Lakes are a vital component of global ecosystems, storing the largest reserves of terrestrial surface liquid water and serving as a cornerstone of water cycle on the Earth^1–3^. They provide essential resources for both human societies and natural ecosystems^4^. The Mongolian Plateau (MP), a fragile transitional zone between arid and semi-arid climates, is one of the regions most vulnerable to global climate change^5,6^. Lakes across the MP are critical water resources that sustain livelihoods, industrial activities, and agricultural production^7,8^. In this ecologically fragile region, lakes serve as a cornerstone of regional economic development while playing a pivotal role in maintaining ecological stability, underscoring their dual importance in both economic and environmental domains^9,10^. However, in recent decades, the ecological environment of the MP has undergone significant degradation, primarily driven by intensified human activities and regional climate change^8,11,12^. This degradation jeopardizes the critical economic and ecological roles of lakes, as evidenced by lake shrinkage, water quality decline, and lake salinization^1,7,13–15^.

Remote sensing technology has become an essential tool for monitoring lake dynamics, particularly at large scales and over extended time periods. In recent years, research on lake dynamics monitoring on the Mongolian Plateau has gradually increased, with a primary focus on two aspects: lake area and water quality^7,10,16–18^. With respect to lake area monitoring, a more systematic understanding of the spatial distribution and temporal evolution of lakes on the Mongolian Plateau has been achieved through the integration of multi-source satellite data, including MODIS, Landsat, and Sentinel imagery^7,8,10,11^. For instance, Tao used satellite remote sensing to document a rapid decline in lake area from the 1980s to 2010, particularly in Inner Mongolia^7^. However, Zhou’s analysis of an extended time series reveals a marked recovery in lake extent since 2009^10^. Despite these insights, there remains considerable debate over the episodic nature of lake area dynamics. This underscores the urgent need for sustained, high-precision, multi-source remote sensing monitoring to comprehensively unravel their long-term evolution. In contrast, remote sensing of lake water quality encompasses a wider range of parameters, including *Secchi Disk Depth* (SDD), *Total Suspended Matter* (TSM), and *Forel–Ule Index* (FUI). Supported by *in situ* observations, researchers have developed inversion models for lake water quality parameters using empirical models and machine learning techniques, offering cost-effective solutions for large-scale water quality monitoring. For example, Zhang estimated SDD of lakes in Inner Mongolia from 1986 to 2018, revealing an overall increasing trend with an average annual improvement of 0.14 meters^19^. Similarly, Tao examined the long-term spatiotemporal dynamics of TSM in Chinese lakes from 1990 to 2020, showing a decline in TSM levels in Inner Mongolia’s lakes, with water quality improving after 2004^16^. However, existing studies have predominantly focused on Inner Mongolia, and a comprehensive understanding of water quality changes across the entire Mongolian Plateau is still lacking.

Although previous studies have revealed the spatiotemporal evolution of lake area and water quality in response to climate change and human activities, integrated analyses that simultaneously consider both aspects remain limited. This limitation hinders a comprehensive assessment of changes in lake water resources. Furthermore, the availability of long-term, publicly accessible datasets encompassing both lake area and water quality across the Mongolian Plateau remains insufficient. While several global lake water quality monitoring efforts have included portions of the region, these studies have primarily concentrated on a limited number of large lakes, and publicly available data products are restricted^20,21^. The lack of consistent temporal and spatial data coverage poses significant challenges for conducting comprehensive regional-scale lake ecosystem modeling and analysis. To overcome this limitation, we developed a high-precision, unified spatiotemporal-scale integrated dataset that merges lake area and water quality dynamics, filling critical spatiotemporal gaps and providing a solid foundation for comprehensive evaluation of lake water resources.

This study aims to provide a comprehensive understanding of the hydrological and water quality dynamics of lakes (including artificial reservoirs) on the MP through long-term remote sensing satellite observations. To achieve this objective, we developed a robust monitoring framework that identified the spatial distribution and maximum extents of 1,161 lakes on the MP while also providing annual spatial locations and polygon boundaries for all lakes. Additionally, we generated annual time series data of three water quality variables including SDD, TSM, and FUI. These variables offer critical information for assessing long-term lake changes and enhance stakeholder access to integrated information on both water quantity and quality. For the first time, this study provides a spatially and temporally consistent dataset at the 30-m spatial resolution and spanning from 1990 to 2020, which is expected to establish a solid data foundation for annual trajectory of MP lake dynamics and to provide new insights into lake ecosystem changes in arid and semi-arid regions.

## Methods

### Study area

**Fig. 1:**
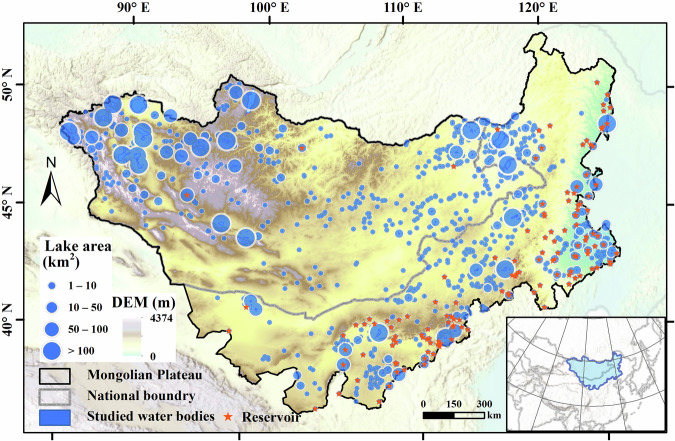
Distribution of lakes with water surface area > 1 km^2^ on the Mongolian Plateau. Inset map shows the spatial location of study area.

The Mongolian Plateau, located in northeastern Asia (37.25°–53.10°N, 87.50°–126.07°E), is bounded by the Greater Khingan Range to the east, the Altai Mountains to the west, the Sayan, Khentii, and Yablonovyy Ranges to the north, and the Yin Mountains to the south (Fig. 1). Its core region, spanning approximately 2.75 million km², includes the Inner Mongolia Autonomous Region of China and Mongolia, with an average elevation exceeding 1,500 meters^22,23^. The plateau features significant topographical variation, characterized by a west-to-east decline in elevation and a diverse geomorphological landscape comprising mountains, hills, plains, plateaus, Gobi deserts, and arid regions^24,25^. These unique geological conditions have fostered numerous lakes, which are vital for sustaining the regional ecosystem and supporting the development of nomadic civilizations, including several large lakes exceeding 1,000 km², such as Uvs, Khovsgol, Hulun, Khyargas, and Khar–Us. In recent decades, intensified human activities and climate change have led to significant lake shrinkage and desiccation on the MP^26^. This has exacerbated environmental degradation, posing severe threats to local livelihoods^7,10^. As the degradation of lakes and grasslands continues, the MP has become a major source of dust storms affecting northern China^13^.

### Study data sources

In this study, we utilized global surface water occurrence data to map the annual dynamics of lakes extents on the MP, while also providing spatial constraints for subsequent analyses of water quality changes. Currently, two widely used surface water occurrence datasets are provide for public, including the *Global Surface Water* (GSW) dataset (https://global-surface-water.appspot.com/) and the *Global Land Analysis and Discovery* (GLAD) laboratory (https://glad.umd.edu/dataset/global-surface-water-dynamics). The GSW dataset^27^, based on Landsat imagery, provides multi-layer information on the spatial distribution and temporal dynamics of global water bodies since 1984, including water presence, disappearance events, and maximum coverage extent. Its annual water extent data offer precise support for monitoring lakes, rivers, and other water bodies. However, given the diverse terrains of the MP, the accuracy of the GSW dataset may be insufficient under certain environmental conditions. To ensure the completeness and precision of extracted lakes, we therefore introduced the GLAD dataset as a supplementary source. The GLAD dataset excels in identifying and monitoring small-sized and morphologically complex water bodies^28^, particularly in high-latitude regions, narrow river corridors, and mountainous areas. By integrating the GSW data with the GLAD dataset—used to fill record gaps (due to cloud cover, sensor errors, or other factors)—we assembled a comprehensive, high-accuracy lake-water distribution dataset for the MP, which provides robust support for fine-scale long-term analyses of lake dynamics in the region.

Dynamic water quality parameters for lakes on the MP are primarily derived through the remote sensing inversion of empirical or semi-empirical models. In this study, we employed the Landsat Collection 1 Tier 1 surface reflectance (SR) and top-of-atmosphere (TOA) datasets provided by the United States Geological Survey (USGS) to monitor lake dynamics across the MP from 1990 to 2020. This dataset comprises imagery from the Landsat 5 Thematic Mapper (TM; 1984–2012), Landsat 7 Enhanced Thematic Mapper Plus (ETM + ; 1999–present), and Landsat 8 Operational Land Imager (OLI; 2013–present). All Landsat imagery was acquired via the *Google Earth Engine* (GEE) platform (https://earthengine.google.org/), a cloud-based system offering high-performance geospatial analysis and an extensive repository of remote sensing datasets^29,30^. Considering the ice-covered period of MP lakes in winters, we selected imagery acquired between May and October, totaling approximately 95,000 scenes. SR products from Landsat 5 and 7 were atmospherically corrected using the Landsat Ecosystem Disturbance Adaptive Processing System (LEDAPS), while Landsat 8 data were corrected using the Landsat Surface Reflectance Code (LaSRC)^31^. To ensure spectral consistency among the various Landsat sensors, the calibration coefficients proposed by Roy *et al*.^32^ were applied, recalibrating OLI reflectance to match those of TM and ETM + . For each image, the cloud, cloud shadow and snow pixels were removed from imagery using CFMask flags embedded in the quality-assurance band, which works well and is suitable for preparing Landsat data for change detection^33,34^. For Landsat 7 ETM + scenes affected by the Scan Line Corrector failure (SLC-off), scan-line gaps were masked, and annual lake boundaries and water-quality metrics were derived from May–October multi-scene medians using only valid pixels. To improve computational efficiency, approximately 56,000 images with less than 30% cloud cover in the study area were retained, ultimately generating a multispectral, cloud-free composite image dataset across lakes on the MP (MPLakeImagery).

### Research framework

**Fig. 2:**
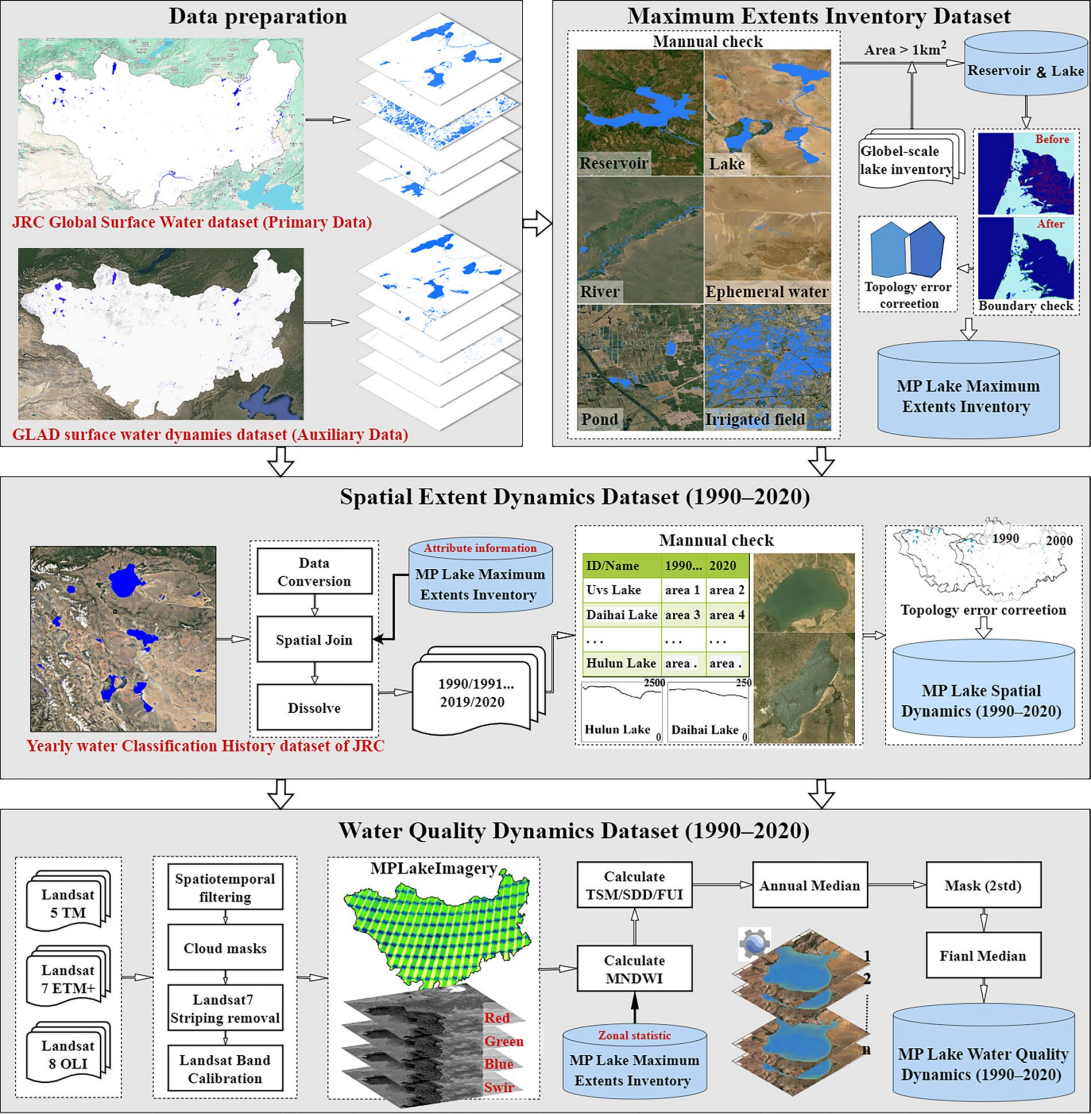
Workflow and Methods for the Generation of the Mongolian Plateau Lake Dataset.

The Mongolian Plateau Lake Dataset (MPLD) was developed through a comprehensive workflow (Fig. 2) that integrates lake surface area data from the GSW dataset, refined through manual inspection, and further combined with the GLAD dataset. This process yielded the maximum extents inventory, encompassing lakes larger than 1 km² across the MP. Building upon this inventory, we derived two key datasets: (1) Mongolian Plateau Lake Spatial Extent Dynamics Dataset (1990–2020) which tracks long-term changes in lake surface area, and (2) Mongolian Plateau Lake Water Quality Dynamics Dataset (1990–2020), which utilizes remote sensing empirical models to estimate critical water quality indicators, including SDD, TSM, and FUI. Together, these datasets provide a systematic, multi-decadal assessment of lake dynamics and water quality variations across the MP. They serve as a foundational resource for investigating the complex interplay between hydrological processes, ecological shifts, and anthropogenic influences on the MP.

### Mongolian Plateau Lake Maximum Extents Inventory Dataset

To enable high-precision, long-term lake monitoring across the MP, we generated the Mongolian Plateau Lake Maximum Extents Inventory Dataset, which serves as a critical baseline for both annual water extents extractions and water quality parameter calculation. Our methodology employed the Max Water Extent (MWE) data layer from JRC GSW, which was initially used to identify water bodies larger than 1 km² within the study area. To improve monitoring accuracy and spatial completeness, we integrated the GLAD water dataset as a supplementary data source, particularly for capturing small or morphologically complex water bodies. The initial extraction encompassed all natural lakes, artificial reservoirs, rivers, ponds, irrigated lands, and temporary water bodies, forming the foundation for further refinement.

Identifying natural lakes and artificial reservoirs from a diverse array of surface water bodies presents a significant challenge. To ensure the accuracy and reliability of the inventory data, this study adopted a two-step approach. In the first step, a detailed manual review was conducted using high-resolution imagery in ArcGIS Pro. Through systematic visual interpretation, rivers, ponds, irrigation areas, and temporary water bodies were excluded, ensuring that only natural lakes and artificial reservoirs were retained for further analysis. Simultaneously, to accurately delineate water body boundaries, the outlines of all water bodies were manually refined, with particular emphasis on artificial reservoirs. This ensured the correction of potential errors and the precise definition of maximum water extents, thereby substantially improving the dataset’s accuracy. Additionally, to enforce a uniform river–lake boundary across the MP, we adopted a single rule set based on the JRC GSW Max Water Extent (MWE ≥ 1 km²). We removed channel-like slivers and micro-fragments ( < 0.01 km²), classified inlet/outlet waters by storage-dominated versus through-flow morphology, excluded adjacent wetlands and ephemeral inundation, and clipped annual extents to each lake’s MWE for temporal consistency. In the second step, the inventory generated in this study was cross-validated against with existing global-scale lake inventory such as HydroLAKES and GeoDAR. Lake and reservoir boundaries were compared to identify and resolve any omissions or inconsistencies, ensuring completeness. Additionally, rigorous topological consistency checks were performed to further reinforce the reliability of the data. The finally result is a high-quality, fully validated catalog of the maximum water extents for natural lakes and artificial reservoirs across the MP, providing a robust foundation for dynamic lake monitoring, water quality analysis, future ecological assessments, and regional water resource management. –

### Mongolian Plateau Lake Spatial Extent Dynamics Dataset (1990–2020)

The Yearly Water Classification History dataset of JRC^27^, generated using 4,716,475 scenes of Landsat 5, 7, and 8 acquired between 16 March 1984 and 31 December 2021 (last update), depicts the temporal and spatial distributions of surface water from 1984 to 2021, with each pixel individually classified as water or non-water by an expert system. In the current study, to effectively capture the long-term dynamics of lakes on the MP, we adopted this dataset to obtain the Seasonal water extent of lakes. By integrating it with the Mongolian Plateau Lake Maximum Extents Inventory Dataset, we precisely extracted the annual spatial distribution of 1,161 lakes and uniformly assigned values to define the spatial extents for each lake across different years. while excluding non-lake water bodies located beyond the catalogued maximum water extents to ensure the accuracy and internal consistency of the final dataset.

**Fig. 3:**
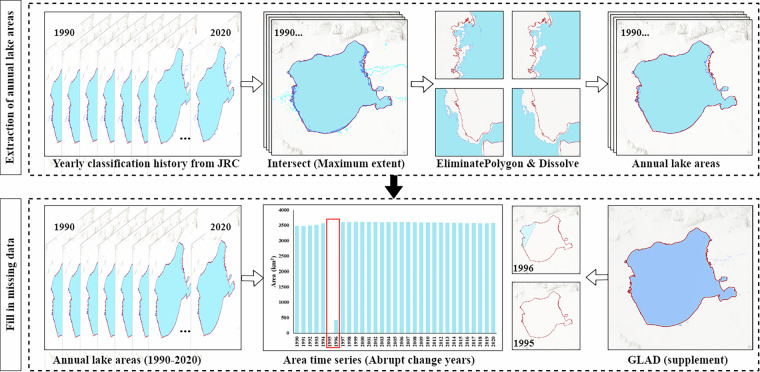
Workflow for annual lake-area mapping (example: Uvs Lake). A two-step workflow comprising (1) spatial extraction and refinement—classifying yearly water with the JRC Yearly Water Classification History and intersecting with the Maximum Water Extent (MWE), then eliminating small fragments and dissolving polygons to obtain coherent annual lake boundaries; and (2) temporal screening and gap filling—building an area time series to flag abrupt-change years, inspecting and correcting anomalies with historical imagery, and filling missing observations using the GLAD surface-water dataset, resulting in a high-quality spatiotemporal record of annual lake areas for 1990–2020.

Additionally, strict quality control procedures were implemented. (1) For internal holes and fragmented boundaries within the same lake, a threshold of 0.01 km² was established, and areas smaller than this threshold were removed. Given the frequent splitting and merging of shallow lakes, particularly in the central and eastern regions of the MP, fragmented patches belonging to the same water body were merged, thereby enhancing the overall accuracy and consistency of the dataset. (2) A time series was generated for each lake. By analyzing these time series, we accurately identified years with abrupt changes in area or missing data. For lakes with abrupt area changes or missing data due to objective factors, historical imagery was manually reviewed to optimize lake boundaries, and the GLAD dataset was used to fill in the gaps. For anomalous years prior to 1999, values from the preceding and succeeding years were used for replacement, ensuring the continuity and completeness of the dataset. The full procedure—illustrated for Uvs Lake—is shown in Fig. 3. Finally, after resolving geometric and topological errors, a comprehensive, rigorously quality-controlled long-term lake spatial distribution dataset for the MP was generated.

### Mongolian Plateau Lake Water Quality Dynamics Dataset (1990–2020)

Using the MPLakeImagery dataset derived from Landsat imagery, this study estimated three key water quality indicators for lakes on the MP: SDD, TSM, and FUI concentration. A comprehensive workflow for processing water quality indicators was developed and implemented. During the preprocessing of the MPLakeImagery dataset, a series of rigorous steps were undertaken, including band renaming, scale factor application, cloud and shadow removal, stripe correction, and band calibration. These procedures resolved discrepancies among satellite images from various sensors, improved the spatial and temporal consistency of the remote sensing data, and facilitated robust spatiotemporal analyses of water quality. For water quality estimation, the Mongolian Plateau Lake Maximum Extents Inventory Dataset was used to provide spatial constraint of lake extent. A *modified normalized difference water index* (MNDWI) was calculated to generate water masks for each imagery, effectively eliminating signal noise from terrestrial and non-water pixels within the maximum water extent. The MNDWI threshold was set to 0, with pixels having MNDWI values above this threshold classified as water and those below classified as non-water. The use of MNDWI can filter out floating water body pixels in each image scene, preventing these non-water pixels from causing errors in the calculation of water quality indicators. This approach ensured that all subsequent analyses were confined to water bodies, thereby significantly enhancing the accuracy of water quality assessments.

Water clarity is typically measured by the depth at which a standard black-and-white Secchi disk becomes invisible when lowered into the water, a parameter referred to as SDD^35^. SDD is a critical indicator of the trophic state and water quality of aquatic ecosystems, influenced by suspended matter, algae, and dissolved organic matter^36^. It plays a key role in regulating underwater light availability, thereby influencing aquatic plant growth, organism habitats, and primary productivity^37^. While traditional ship-based sampling methods remain the most accurate and widely used for SDD measurements, they are inadequate for large-scale trend analyses over extended spatial and temporal scales^15^. In recent years, remote sensing technology has been extensively applied for quantitative analysis of SDD dynamics at regional scales due to its broad spatial coverage and efficient data acquisition capabilities^15,38–40^. To estimate the SDD of lakes across the MP region, this study employed an empirical model proposed by Zhang *et al*.^15^, which is based on the red band reflectance (Rs(R)) from the Landsat satellite series. The model exploits the strong correlation between red band reflectance and SDD and has demonstrated reliable accuracy across extensive calibration and validation datasets (R² = 0.73, MRE = 34.2%, NRMSE = 55.4%). Specifically, the model was calibrated using 887 pairs of field-measured SDD data and concurrent Landsat imagery, collected from lakes across various regions in China. Additionally, the model was validated using 246 independent SDD measurements to further test its performance. The model is expressed as a power-law equation:

1 $${SDD}=0.46\times {\left(\frac{{R}_{s}\left(R\right)}{\pi }\right)}^{-1.26}$$

TSM is another critical water quality indicator, acting as a proxy for the ecological health of aquatic ecosystems. It is closely associated with the distribution of nutrients, micropollutants, and heavy metals^41,42^. Traditional methods for measuring TSM rely on field sampling and laboratory analysis. However, their limitations in temporal and spatial coverage hinder their applicability for large-scale and long-term dynamic monitoring^16,43^. To estimate TSM concentrations in lakes across the MP, this study utilized an exponential model proposed by Tao *et al*.^16^, which is based on the ratio of red band reflectance Rs(R) to blue band reflectance Rs(B) from Landsat satellite imagery. The model has been extensively validated using a large field measurement dataset, demonstrating strong performance with R² = 0.87, RMSE = 10.16 mg/L, and MAPE = 38.37%. Specifically, the model was calibrated using 338 match-ups of *in-situ* measured TSM data and Landsat TOA reflectance, and validated using 169 match-ups. This extensive validation dataset provides a solid foundation for confirming the model’s reliability and applicability. The model is expressed as follows:

2 $${TSM}=0.0933\times {e}^{8.0047\left(\frac{{R}_{S}\left(R\right)}{{R}_{s}\left(B\right)}\right)}$$

Water color plays a critical role in traditional water quality assessments^44^. To standardize the measurement and representation of water color, the Commission International DE L’Eclairage (CIE) developed the CIE XYZ color system^45^, which is widely used for calculating the FUI of water color. In this process, visible light RGB bands are first converted into the CIE-XYZ tristimulus values X, Y, and Z. These tristimulus values are then normalized to derive the chromaticity coordinates (x, y) in the CIE chromaticity system. Using the chromaticity coordinates, the chromaticity angle α is calculated and further corrected with a polynomial function^46^. The final FUI value is determined by referencing a look-up table that maps α to corresponding FUI levels. In Landsat imagery, the red, green, and blue bands correspond to Rs(R), Rs(G), and Rs(B), respectively.

3 $$\begin{array}{c}\alpha =\left(\arctan 2\frac{x-0.3333}{y-0.3333}\right)\frac{180}{\pi },x=\frac{X}{X+Y+Z},{y}=\frac{Y}{X+Y+Z}\\ X=2.7689\times {R}_{s}\left(R\right)+1.7517\times {R}_{s}\left(G\right)+1.1302\times {R}_{s}\left(B\right)\\ Y=1.0000\times {R}_{s}\left(R\right)+4.5906\times {R}_{s}\left(G\right)+0.0601\times {R}_{s}\left(B\right)\\ Z=0.0565\times {R}_{s}\left(G\right)+5.5943\times {R}_{s}\left(B\right)\end{array}$$

4 $$\varDelta =-84.94{a}^{5}+594.17{a}^{4}-1559.86{a}^{3}+1852.50{a}^{2}-918.11a+151.49\left(a=\frac{\alpha }{100}\right)$$

**Fig. 4:**
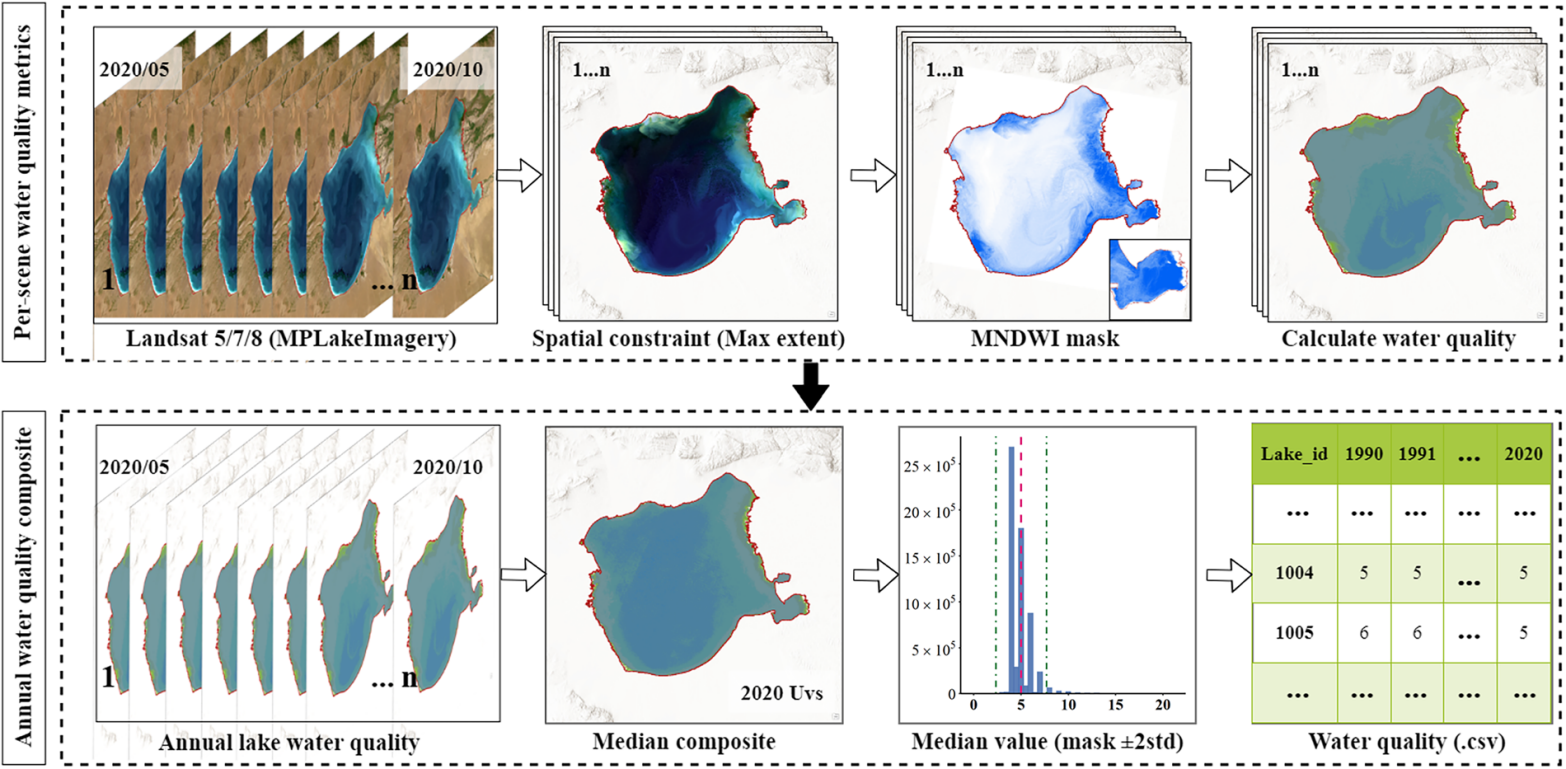
Workflow for annual lake–water-quality indicators (example: Uvs Lake). A two-step workflow comprising (1) spatial masking and constraints—applying the lake’s Maximum Water Extent (MWE) and computing MNDWI to generate per-scene water masks for the preprocessed MPLakeImagery stack; and (2) per-scene retrieval and annual aggregation—computing SDD and TSM for each Landsat 5/7/8 scene, removing outliers with a ± 2-standard-deviation filter, and deriving annual values via median compositing with results exported as a lake-year table (.csv) for long-term monitoring.

Based on the water-quality calculation methods described above, the processed MPLakeImagery dataset was used to compute scene-level water-quality indicators for each image. For each lake–year, we used all available Landsat 5/7/8 scenes acquired within the May–October ice-free window; scenes were restricted to open water using the lake’s Maximum Water Extent (MWE) and per-scene MNDWI masks, and were screened for cloud/snow/shadow prior to retrieval. Note that the per-scene MNDWI step can occasionally identify scene-specific temporary water pixels within the MWE (e.g., ephemeral shoreline inundation driven by seasonal water-level changes). These “scatter-like” polygons are not part of the permanent lake boundaries. Annual parameters were then derived by median compositing the remaining per-scene retrievals after excluding outliers outside mean ± 2 standard deviations. This outlier screening combined with median compositing effectively suppresses transient detections so they do not affect the final maps, statistics, or vector outlines. Years with insufficient valid scenes were flagged and excluded from trend analyses. All resulting annual SDD and TSM series were exported as a lake–year CSV for downstream analysis and applications. The complete processing workflow—using Uvs Lake as an exemplar—is depicted in Fig. 4. By employing a standardized preprocessing workflow and robust indicator-calculation methods, this study enables efficient and reliable long-term monitoring of lake water quality across the MP. The integration of Landsat imagery, remote-sensing models, and rigorous quality control ensures the accuracy and consistency of the dataset and provides valuable insights into spatiotemporal water-quality dynamics across diverse lake ecosystems on the MP.

## Data Records

The Mongolian Plateau lake dataset (MPLD) is publicly accessible through the *figshare* repository (10.6084/m9.figshare.30018049)^47^. The dataset is organized into two main directories. The first directory, “*Data Value File*”, contains three subdirectories: “*Lake maximum extents inventory SHP*”: This subdirectory stores the inventory data for the maximum water extents of lakes. “*Annual Water body dynamic range SHP*”: This subdirectory contains the annual dynamic range data of lake water extents. “*Annual water quality CSV*”: This subdirectory includes statistical data on lake areas and water quality from 1990 to 2020. The second directory, “*Supplement*”, contains validation data for lake areas and water quality, as well as scripts for calculating water quality indicators, implemented on the GEE platform. All vector data in the dataset are provided in ESRI Shapefile format and referenced to the EPSG:4326 (WGS 1984) spatial coordinate system. The vector file for the inventory of maximum water extents includes the following attributes: Code, Perimeter, Area, Longitude, Latitude, Reservoir, Country, Name, and a unique identifier (lake_id). Detailed attribute descriptions are provided in Table 1.

**Table 1:** Data labels and descriptions for the shapefiles.

Attribute	Description and values
shape	Feature type of the lake object.
lati	Latitude of the dam point in decimal degree (type: float) on datum World Geodetic System (WGS) 1984.
longi	Longitude of the dam point in decimal degree (type: float) on WGS 1984.
perimeter	Perimeter of the lake polygon in kilometer.
poly_area	Area of the lake’s water surface in square kilometer.
name	English name of the lake. Some lakes may have alias.
country	The name of the country where the lake is located in.
province	The name of the province where the lake is located in.
lake_id	Identifies each lake with lake inventory dataset.
reservoir	1 represents that the lake is a reservoir; 0 represents that the lake is not a reservoir.
notes	Statements for specific cases in English, e.g. two lakes merged to one single lake due to drastic expansions.

## Technical Validation

### Cross-evaluation of the lake-surface area with manually digitized data

**Fig. 5:**
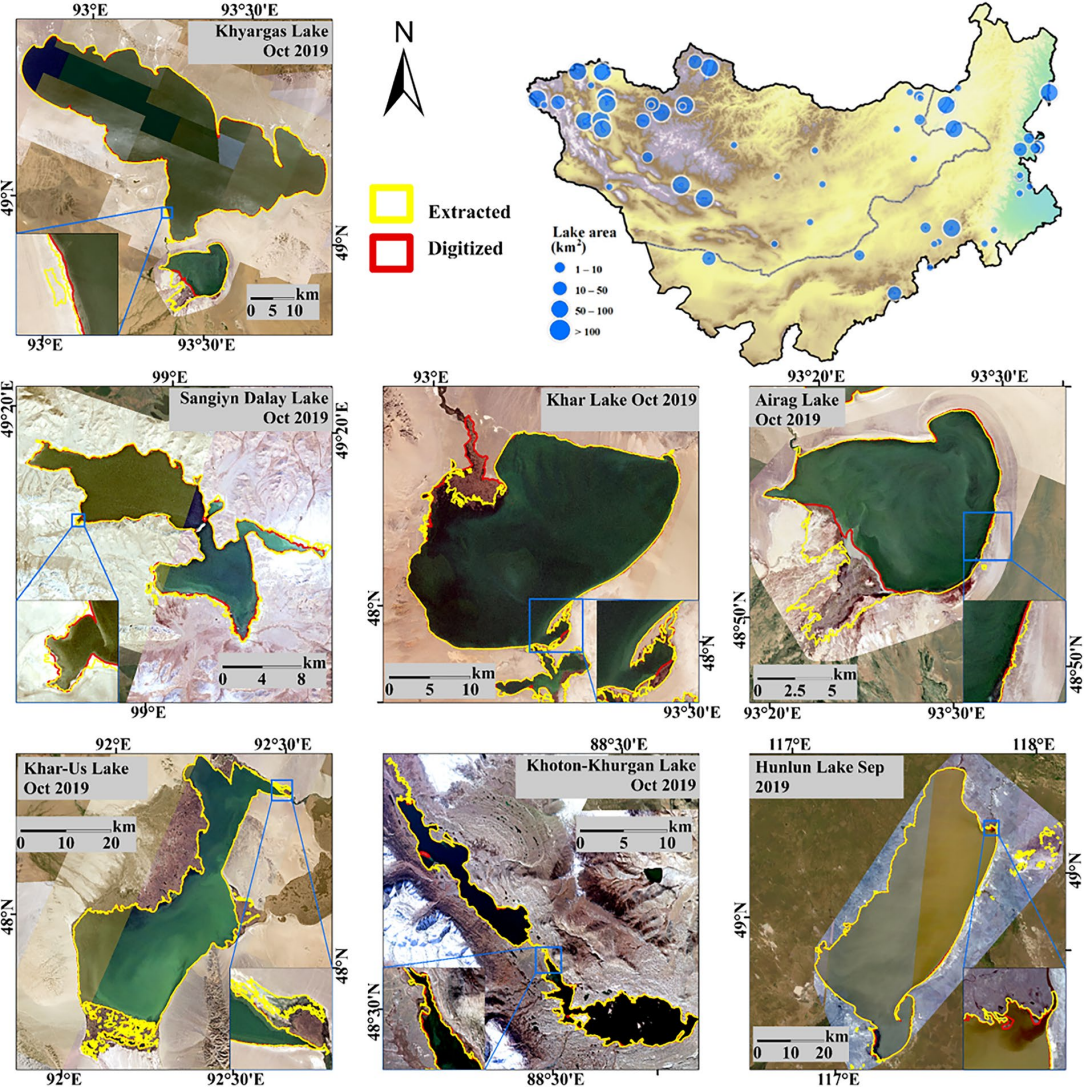
Validation of Lake Surface Area: The yellow lines represent the water extent derived from the MPLD, while the red lines indicate the water extent identified through Planet satellite imagery. The panels provide examples of randomly selected lakes with varying sizes and characteristics, highlighting the areas where errors occur during validation. The primary errors are found in regions where small inflowing rivers and temporary water bodies are misclassified as part of lakes. The top-right map illustrates the geographic distribution of the sampled lakes used in the validation process.

**Fig. 6:**
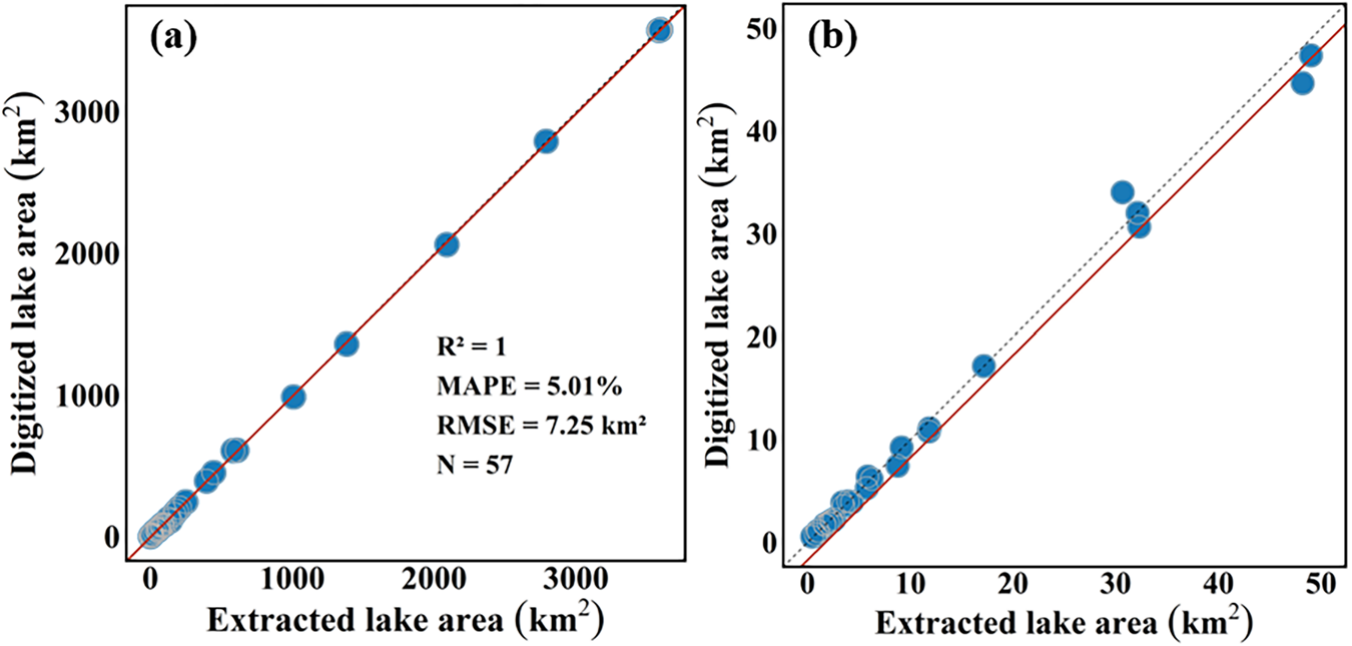
Accuracy Assessment of Extracted Lake Areas in the MPLD: (**a**) Scatter plot showing the relationship between the extracted lake areas and the digitized lake areas. The low mean absolute percentage error (MAPE = 5.01%) validate the high accuracy of the MPLD. (**b**) Zoomed-in scatter plot focusing on lakes with an area smaller than 50 km², demonstrating the consistency of the extracted and digitized lake areas even for smaller water bodies.

To validate the accuracy of the extracted lake area dataset, a systematic validation was performed between lake areas derived from the GSW dataset and those obtained from high-resolution remote sensing imagery. The high-resolution PlanetScope CubeSat satellite imagery was sourced from Planet (https://www.planet.com), which provides optical images at a spatial resolution of approximately 3 meters. These high-resolution images were used to generate reliable reference data and manually delineate lake boundaries, ensuring robust support for the validation process. To ensure representativeness, lakes of various sizes and from different regions were randomly selected as validation samples (Fig. 5). For each lake, the lake extent was visually interpreted using CubeSat imagery and compared with the corresponding lake area extracted from the GSW dataset. The results demonstrated a strong consistency between the two datasets, with an overall bias of less than 5% (Mean Absolute Percentage Error (MAPE) = 5.1%, Root Mean Squared Error (RMSE) = 7.25 km²) and even smaller bias for lakes larger than 50 km² (Fig. 6). These validation confirmed the reliability of the dataset for both static and dynamic lake area monitoring, underscoring its applicability for long-term hydrological studies.

Despite the overall high accuracy of the dataset, some errors remain. The primary errors occur in areas where small inflowing rivers and temporary water bodies are misclassified as part of lakes (Fig. 5). These issues arise from the use of the Yearly Seasonality Classification collection of the GSW dataset, which is designed to comprehensively capture regional water dynamics. Additionally, the extracted lake areas were generally found to be slightly larger than manually digitized data (Fig. 6). This discrepancy is primarily due to the dataset’s tendency to include adjacent wetlands or small water bodies, a phenomenon that closely aligns with actual conditions. To mitigate these errors, rigorous quality control was implemented during the compilation of historical lake dynamics. High-resolution historical imagery was employed to identify and correct misclassified areas, while manual adjustments were conducted using the GLAD dataset to refine boundary accuracy. Boundary deviations were also noted in regions with complex boundaries or significant mixed pixels. These deviations are largely attributed to differences in data resolution: manually digitized results rely on sub-meter resolution imagery, whereas the GSW dataset is based on 30-meter resolution Landsat imagery. Although these deviations exist, they are reasonable and acceptable within the scope of regional-scale lake monitoring.

### Water Quality Validation

Following the validating the lake area dataset for the MP, we further assessed the uncertainty of water quality parameters, with a focus on two key indicators: SDD and TSM. This validation involved a detailed correlation analysis between *in-situ* measurement data from lakes in Inner Mongolia and remote sensing-derived SDD and TSM results. The primary goal was to assess the reliability and accuracy of retrieval methods for water quality monitoring across the region. The validation data used in this study were primarily sourced from the Nanjing institute of Geography and Limnology and Northeast Institute of Geography and Agroecology, Chinese Academy of Sciences. Between 2013 to 2020, the research team conducted field campaigns across 19 lake systems in Inner Mongolia, collecting key water quality parameters, including TSM and SDD. TSM concentrations were determined in the laboratory using Whatman GF/F filters, while SDD was measured in the field using a Secchi disk. These data provide critical baseline information for understanding the ecological status and the impacts of environmental change. We additionally benchmarked our results against NOAA CoastWatch’s Kd(490) product^48^ and the global TSS remote-sensing dataset reported by Jiang *et al*.^49^. Figure S1 shows close spatial agreement between our retrievals and both references, demonstrating strong consistency and broad applicability across diverse lake types.

To ensure consistency between remote sensing data and field measurements, satellite images were carefully selected to match the geographic locations of the *in-situ* measurements. A 3 × 3-pixel window was applied to extract the average reflectance values, ensuring that the extracted data represented the lake surface conditions accurately. The temporal window for image acquisition was also strictly controlled, with satellite image acquisition dates limited to within ± 7 days of the field campaigns. This matching approach has been demonstrated to be reasonable and effective in producing reliable results^16,50^. Although field data were only available for lakes in Inner Mongolia, the region shares substantial climatic and hydrological similarities with other parts of MP, making it a representative area for assessing broader water quality characteristics across the plateau^10,51^.

**Fig. 7:**
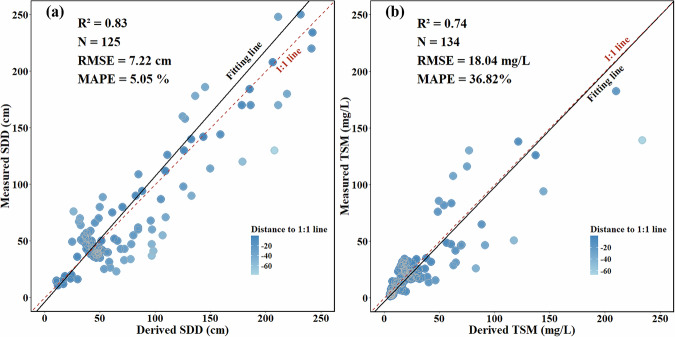
Validation of TSM and SDD Models: This figure compares measured and derived values for TSM and SDD models, with the fitting line (black) and 1:1 line (red dashed) for reference. The color gradient represents the distance to the 1:1 line, showing deviations between measured and derived values. (**a**) Validation of the SDD model, with an R² of 0.83, RMSE of 7.22 cm, and MAPE of 5.05%. (**b**) Validation of the TSM model, with an R² of 0.74, RMSE of 18.04 mg/L, and MAPE of 36.82%.

As illustrated in the Fig. 7, the selected models demonstrated robust performance in validating water quality parameters for lakes across the MP. For SDD, a total of 125 sample pairs were collected and analyzed. The comparison between *in situ* measurements and remote sensing-derived values showed a high level of agreement, with R² = 0.83, RMSE = 7.22 cm, and MAPE = 5.05%. These metrics indicate that the remote sensing method achieves high accuracy in estimating SDD. For TSM, 142 sample pairs were analyzed. The results also revealed a solid correlation between measured and remote sensing-derived TSM values, with R² = 0.74, RMSE = 18.04 mg/L, and MAPE = 36.82%. While the TSM model exhibits relatively higher variability compared to the SDD model, its performance remains adequate for practical monitoring applications. Overall, the findings validate the robustness and applicability of the remote sensing models for estimating water quality parameters in lakes across the MP. These results provide a reliable basis for long-term water quality monitoring and sustainable management of lake ecosystems in the region.

**Fig. 8:**
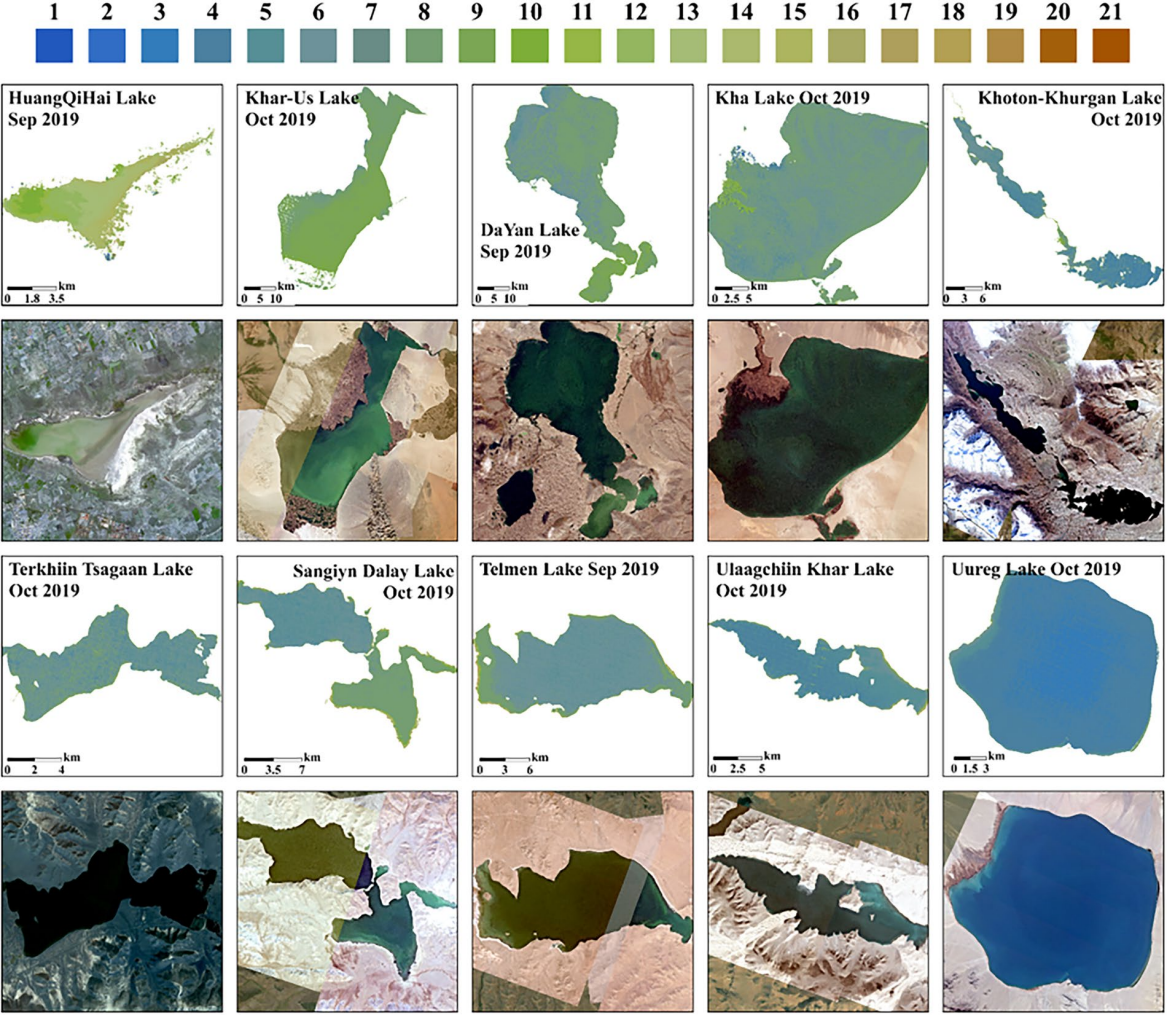
Satellite Imagery of Lakes with FUI Water Color Classifications and Spatial Variation Analysis, Planet satellite imagery of lakes with diverse surface areas, visualized using Bands 1, 2, and 3, alongside water color classifications based on the FUI.

Figure 8 presents the classification maps of lake water color based on the FUI across different area scales. The figure integrates true-color CubeSat imagery (Bands 1, 2, and 3) with corresponding FUI-derived classifications to visualize spatial variations in water color. Ranging from 1 (blue) to 21 (brown), the FUI effectively captures spatial variations in water color both between lakes and within individual lakes. By comparing FUI classifications with high-resolution true-color imagery, the figure provides a qualitative assessment of the FUI’s reliability in representing water color variations. The results demonstrate a strong consistency between FUI-derived water color maps and the visual patterns observed in true-color imagery. The FUI accurately captures spatial heterogeneity in water color, reflecting intra-lake differences influenced by factors such as sediment load, chlorophyll concentration, and dissolved organic matter. Furthermore, distinct FUI gradients within individual lakes align closely with visible color variations in satellite imagery, confirming the FUI’s sensitivity to detecting subtle differences in water quality.

**Fig. 9:**
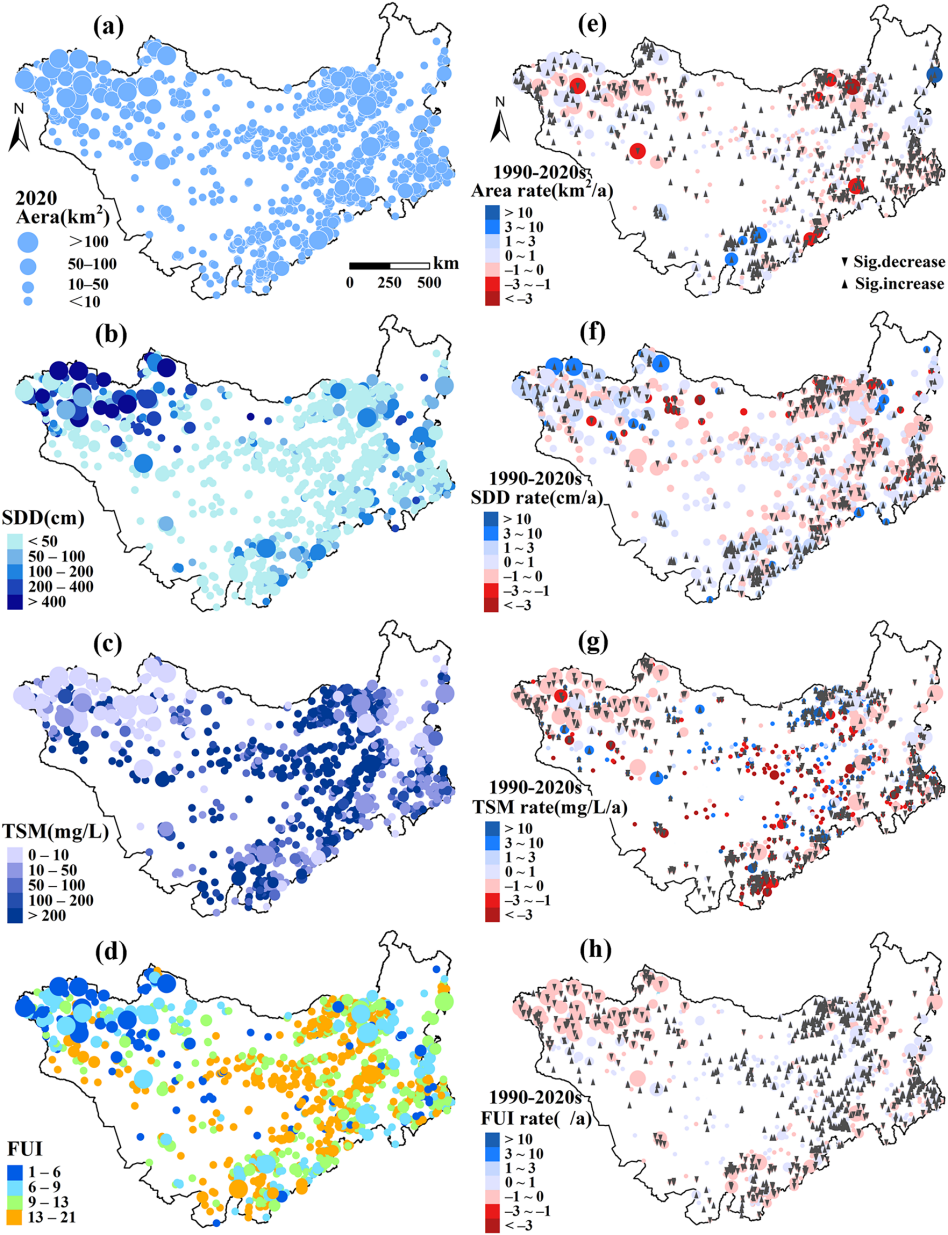
Spatiotemporal dynamics of lake area and water-quality indicators on the Mongolian Plateau (1990–2020). (**a**) maps the lake-surface area in 2020; (**b**) depicts SDD; (**c**) illustrates TSM; (**d**) shows FUI. Panels (**e**)–(**h**) trace the temporal trajectories of lake-surface area, SDD, TSM, and FUI, respectively, revealing pronounced spatial heterogeneity and distinct region-specific trends in lake evolution and water quality.

## Usage Notes

The expansion of lake area does not necessarily indicate an improvement in ecological conditions, as factors such as pollution, sediment accumulation, and water quality degradation may offset the potential ecological benefits of increased water extent. Consequently, focusing solely on lake area may overlook the broader complexity of ecological status and water quality dynamics in lakes. The release of the MPLD bridges the monitoring gap for small lakes ( < 10 km²) and addresses the limitations of previous studies that relied on interval-based analyses with limited temporal continuity. By enabling integrated, long-term monitoring of hydrological and water quality changes across the MP, the dataset offers critical insights into lake dynamics over time (Fig. 9). It supports comprehensive analyses of lake change processes, facilitates the precise identification of ecologically vulnerable lakes, and provides an essential scientific foundation for the development of policies related to lake conservation, restoration, and sustainable management. The inventory includes both natural lakes and artificial reservoirs; a binary reservoir flag (1 = reservoir; 0 = natural lake) enables type-specific filtering when lake-only or reservoir-only analyses are desired.

The long-term time series monitoring results from the MPLD can be integrated with climate change data and human activity records to enhance the understanding of the driving mechanisms behind lake changes. At the regional scale, the MPLD facilitates comparative analyses of lake dynamics within the same watershed, providing valuable insights into variations in lake size and water quality changes. Such cross-lake interconnectivity analyses are essential for developing systematic, watershed-based lake management and water quality protection strategies. By leveraging the MPLD, trends in lake changes across regions offer a solid scientific foundation for localized water resource management. Furthermore, the dataset’s long-term monitoring capabilities enable the validation of lake ecosystem degradation or recovery under the combined effects of climate change and human activities. This dataset plays a pivotal role in promoting the health of lake ecosystems and advancing water resource conservation efforts.

### Limitations, and Future Development of the Dataset

**Fig. 10:**
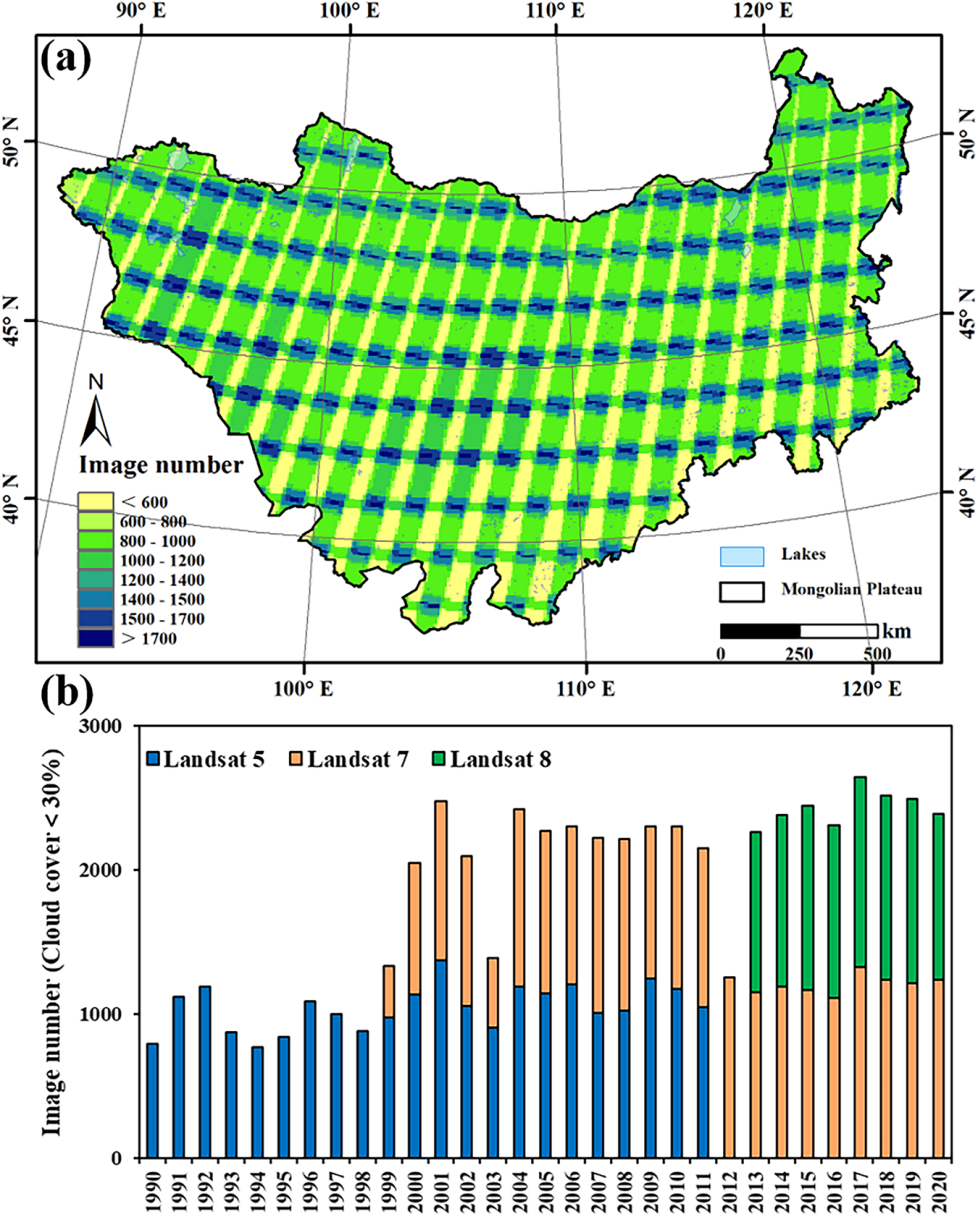
Spatiotemporal distribution of high-quality Landsat data availability (Cloud Cover < 30%) over the Mongolian Plateau from May to October, 1990–2020. (**a**) Spatial map of cumulative Landsat image counts per pixel; (**b**) Annual counts of cloud-free (cloud cover < 30%) scenes by sensor (Landsat 5, 7, 8).

This dataset was developed using the GSW dataset, supplemented with high-resolution imagery to identify and extract lakes larger than 1 km² on the MP. It provides comprehensive coverage of lake water extent and water quality data from 1990 to 2020. However, several limitations remain. In particular, the lower resolution of early Landsat imagery and the technical constraints of earlier sensors led to incomplete water quality records for some lakes, particularly before 2000. The limited availability of Landsat images for surface water mapping in the 1990s may have hindered the accurate capture of annual lake dynamics in certain years. To address this, we supplemented incomplete data by incorporating mapping results from neighboring years. Figure 10 provides a visual representation of annual scene counts and cloud-free coverage ratios, illustrating the challenges in data availability, particularly in the earlier years of the study. Notably, the launch of the Landsat 7 ETM + satellite in 1999 significantly improved the frequency and quality of Earth observations, greatly enhancing the reliability of lake extent and water quality data in the following years.

This dataset does not include lake water volume data, using lake area changes as a proxy for hydrological dynamics. While fluctuations in lake area are widely accepted as an effective indicator of water resource trends, they do not directly reflect lake water volume. Methods for estimating lake volume based on statistical relationships with measurable parameters^52–54^, such as lake area and shoreline buffer slope, are well-established but depend on accurate depth data, which is lacking for most lakes in the region. This gap introduces uncertainty in volume estimates. We call for enhanced research collaboration to conduct comprehensive water volume surveys of medium- and large-sized lakes, improve depth data availability, and ultimately support more reliable water volume estimates. With better volume data, future studies will be able to provide a more complete analysis of lake water resources, supporting more accurate regional water resource management and decision-making.

Looking ahead, we will explore multi-band machine-learning retrievals to improve parameter accuracy once expanded *in-situ* datasets are available. This will require strengthened international collaboration and standardized field campaigns across the MP to increase the volume and geographic balance of training data. With sufficient, well-stratified measurements, we will benchmark ML against the current approach and report accuracy and uncertainty transparently; until then, we prioritize a transparent, scalable workflow that ensures plateau-wide consistency and reproducibility.

## Supplementary information

Supplementary Materials for SDATA-25-02951A

## Data Availability

All data supporting the findings of this study are openly available in the *figshare* repository at 10.6084/m9.figshare.30018049^47^. Further details on file lists, file types, folder structure, and variable/column names are provided in the Data Records section.

## Code availability

The data-production algorithms were implemented on the GEE platform using water-index methods. The scripts used to calculate water-quality indicators and reproduce the core processing are provided within the dataset’s “*Supplement*” directory at the same *figshare* repository (10.6084/m9.figshare.30018049)^47^.
