# Supplementary material for: A three-decade lake dataset on the Mongolian plateau tracking water area and quality dynamics (1990–2020)
Source: Sci Data. 2025 Nov 14;12:1788. doi: 10.1038/s41597-025-06059-5 (PMC12618646; doi:10.1038/s41597-025-06059-5)
Supplement: Supplementary file 1 — Supplementary Materials for SDATA-25-02951A [file 41597_2025_6059_MOESM1_ESM.pdf]

Supplementary Materials for SDATA-25-02951A

A three-decade lake dataset on the Mongolian plateau tracking water area and quality dynamics (1990 – 2020)

Jinkai Guo[1,2], Kai Liu[2,3*], Jiaming Na[1*], Ge Liu[4], Zhigang Cao[2,3], Chenyu Fan[2], Bin Xue[2,3,5] , Junchuan Huang[6] & Chunqiao Song[2,3,5]

[1]College of Civil Engineering, Nanjing Forestry University, Nanjing 210037, China.

[2]State Key Laboratory of Lake and Watershed Science for Water Security, Nanjing institute of Geography and Limnology, Chinese Academy of Sciences, Nanjing, 211135, China.

[3]University of Chinese Academy of Sciences, Nanjing (UCASNJ), Nanjing, 211135, China.

[4]Northeast Institute of Geography and Agroecology, Chinese Academy of Sciences, Changchun, 130102, China.

[5]University of Chinese Academy of Sciences, Beijing, 100049, China.

[6]Research Center of Applied Geology of China Geological Survey, Chengdu, 610036, China

*Corresponding author: kliu@niglas.ac.cn (K. Liu) & jiaming.na@njfu.edu.cn (J. Na)

To assess transferability and parameter stability, we expanded validation to 19 Inner Mongolian lakes spanning arid–semi-arid climates and a wide optical gradient. We then performed external benchmarks: SDD against NOAA CoastWatch Kd(490) (an inverse clarity proxy) and TSM against the global TSS product of Jiang et al. (2021). As illustrated by four representative lakes in Fig. S1 (Achit Nuur, Khar-Us, Khyrgas–Airag, Uureg Nuur), large-scale structures are consistent across products: high Kd(490) co-locates with low SDD, and major suspended-matter features (nearshore belts, channel plumes) captured by TSS are reproduced by our TSM maps. Residual differences are localized and explainable—primarily in optically shallow margins (bottom reflectance), in coarse-grid Kd(490) cells that smooth shoreline gradients, and in very high-backscatter plumes where red-band responses compress.

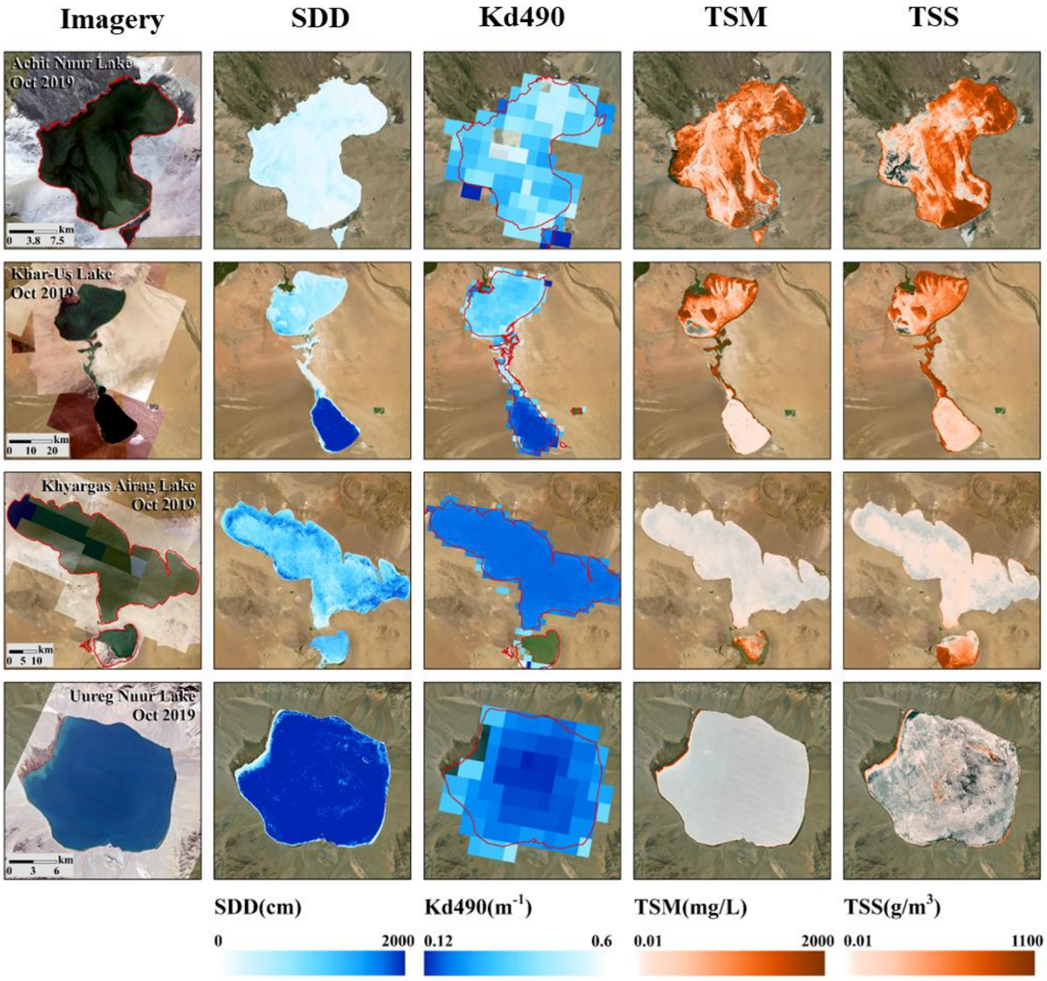

Fig. S1 Spatial distribution of water quality parameters in Mongolian Lakes. Satellite imagery and water quality parameters (SDD, Kd490, TSM, and TSS) for four lakes (Achit Nuur Lake, Khar-Us Lake, Khyargas Airag Lake, and Uureg Nuur Lake) from October 2019. The color maps represent variations in water transparency (SDD), optical

properties (Kd490), suspended matter (TSM), and total suspended solids (TSS). SDD and TSM data are from our dataset, Kd490 is sourced from NOAA, and TSS data is based on the global product (Jiang et al., 2021).

Table S1 presents the annual total and cloud-free Landsat scene counts for the Mongolian Plateau from May to October, covering the years 1990 to 2020. The table provides a detailed breakdown of the total number of scenes available each year and the number of cloud-free scenes (cloud cover <30%), which are crucial for accurate water quality assessments. This table supplements the information in Figure 10 and offers a more granular, year-by-year view of image availability and data quality, providing readers with a clearer understanding of the dataset's temporal coverage and the limitations imposed by cloud cover, especially in the earlier years.

Table. S1 Annual total and cloud-free Landsat scene counts over the Mongolian Plateau (May–October 1990–2020)

Year	Total Scenes	Cloud Free Scenes	Landsat 5	Landsat 7	Landsat 8	Cloud Free Ratio
1990	1391	794	794	0	0	0.571
1991	1964	1119	1119	0	0	0.570
1992	1892	1186	1186	0	0	0.627
1993	1538	873	873	0	0	0.568
1994	1333	769	769	0	0	0.577
1995	1419	841	841	0	0	0.593
1996	1729	1087	1087	0	0	0.629
1997	1721	998	998	0	0	0.580
1998	1584	879	879	0	0	0.555
1999	2077	1331	978	353	0	0.641
2000	3449	2043	1131	912	0	0.592
2001	3701	2472	1373	1099	0	0.668
2002	3451	2098	1055	1043	0	0.608
2003	2541	1389	904	485	0	0.547
2004	3941	2423	1186	1237	0	0.615
2005	3724	2269	1138	1131	0	0.609
2006	3721	2298	1208	1090	0	0.618
2007	3444	2218	1009	1209	0	0.644
2008	3620	2213	1024	1189	0	0.611
2009	3635	2303	1243	1060	0	0.634
2010	3360	2301	1175	1126	0	0.685
2011	3459	2150	1047	1103	0	0.622
2012	1918	1250	0	1250	0	0.652

2013	3852	2264	0	1148	1116	0.588
2014	4403	2383	0	1190	1193	0.541
2015	4406	2446	0	1165	1281	0.555
2016	4220	2312	0	1109	1203	0.548
2017	4526	2639	0	1321	1318	0.583
2018	4422	2516	0	1234	1282	0.569
2019	4353	2487	0	1212	1275	0.571
2020	4391	2388	0	1238	1150	0.544
